# Removal of Zinc from Concentrated Galvanic Wastewater by Sodium Trithiocarbonate: Process Optimization and Toxicity Assessment

**DOI:** 10.3390/molecules28020546

**Published:** 2023-01-05

**Authors:** Maciej Thomas, Zuzana Melichová, Matej Šuránek, Joanna Kuc, Angelika Więckol-Ryk, Paweł Lochyński

**Affiliations:** 1 Faculty of Environmental Engineering and Energy, Cracow University of Technology, Warszawska 24, 31-155 Cracow, Poland; 2 Department of Chemistry, Faculty of Natural Sciences, Matej Bel University, Tajovskeho 40, 97401 Banská Bystrica, Slovakia; 3 Faculty of Chemical Engineering and Technology, Cracow University of Technology, Warszawska 24, 31-155 Cracow, Poland; 4 Department of Extraction Technologies, Rockburst and Risk Assessment, Central Mining Institute, 40-166 Katowice, Poland; 5 Institute of Environmental Engineering, Wrocław University of Environmental and Life Sciences, 50-365 Wrocław, Poland

**Keywords:** zinc, sodium trithiocarbonate, coagulation, response surface methodology, phytotoxicity

## Abstract

In the present research, the removal of zinc from concentrated galvanic wastewater (pH 3.1, conductivity 20.31 mS/cm, salinity, 10.16 g/L, Chemical Oxygen Demand (COD) 2900 mg O_2_/L, Total Organic Carbon (TOC) 985 mg/L, zinc (Zn) 1534 mg/L and ethylenediaminetetraacetic acid (EDTA) 70 mg/L) by combination of lime (Ca(OH)_2_) and sodium trithiocarbonate (Na_2_CS_3_) as precipitation agents is studied. Central Composite Design (CCD) and response surface methodology (RSM) were applied for modelling and optimizing the designed wastewater treatment process. Analysis of Variance (ANOVA) and the experimental verification of the model confirmed the consistency of the experimental and estimated data calculated from the model (R^2^ = 0.9173, R^2^_adj._ = 0.8622). The use of Ca(OH)_2_ and Na_2_CS_3_ in the optimal condition calculated from the model (pH = 10.75 ± 0.10, V Na_2_CS_3_ dose 0.043 mL/L and time = 5 min) resulted in a decrease in the concentration of Zn in treated wastewater by 99.99%. Other physicochemical parameters of wastewater also improved. Simultaneously, the application of Ca(OH)_2_ and Na_2_CS_3_ reduced the inhibition of activated sludge dehydrogenase from total inhibition (for raw wastewater) to −70% (for treated wastewater). Under the same conditions the phytotoxicity tests revealed that the seed germination index for the raw and treated wastewater increased from 10% to 50% and from 90% to 100% for white mustard (*Sinapis alba*) and garden cress (*Lepidium sativum* L.), respectively. The parameters of root and shoot growth showed a statistically significant improvement. Treated wastewater (1:10) showed a stimulating effect (shoot growth) compared to the control sample (GI = −116.7 and −57.9 for *S. alba* and *L. sativum* L., respectively). Thus, the use of Na_2_CS_3_ is a viable option for the treatment of concentrated galvanic wastewater containing zinc.

## 1. Introduction

Heavy metals are generally defined as elements with relatively high densities, atomic weights, or atomic numbers, and also as metallic elements that have a relatively higher density compared to pure water [1]. Some of them, e.g., Cr, Cd, Hg, Tl and Ni, are hazardous not only in compounds but also in elemental forms. Heavy metal compounds, especially nitrates, are highly soluble in water and because of that, heavy metal cations are detected in living micro(organisms), e.g., in the muscle tissue, liver and gills of fishes extracted from contaminated waters. It has been proven that heavy metal cations can get into the food chain and then accumulate within human bodies, making them especially dangerous. Many studies have shown that heavy metals and metalloids, e.g., Hg, As, Pb, Cd and Cr, can disturb human metabolomics and contribute to increased morbidity and even mortality [2,3,4]. In addition, heavy metals are non-biodegradable and in many cases can be carcinogenic [5,6,7,8,9,10]. The degree of toxicity of selected heavy metals to humans varies as follows: Co < Al < Cr < Pb < Ni < Zn < Cu < Cd < Hg [11], and the toxic effect to humans depends on many variables such as the type of heavy metal and type of compound, and its solubility, dose, method and time of exposure.

Usually in unpolluted waters, heavy metals occur in trace amounts but are found in larger amounts in industrial wastewater, especially in untreated industrial wastewater. Zinc is one of the more commonly used metals and is utilized as a protective surface for iron/steel and for the manufacturing of zinc alloys (brass), rolled zinc, zinc dust (reducing agent and coloring agent) and zinc compounds such as zinc oxide (vulcanizing, pigment and paint), zinc chloride (flux, electrolyte in dry cell batteries, and corrosion inhibitor in water treatment), zinc cyanide (metal plating, electroplating and gold extraction), zinc fluoride (electroplating baths, in galvanizing steel) and zinc sulfate (paper bleaching, textile dyeing and printing,) [12]. Exposure to large amounts of zinc, even for a short time, can cause stomach cramps, nausea and vomiting. In the long term, exposure to zinc may cause serious health issues that include but are not limited to anemia, pancreas damage and the decreasing of HDL cholesterol; however, zinc in trace amounts is essential for human health [12]. In fact, some heavy metals, Zn, Cu, Fe and Cr(III), are essential components for biochemical processes in living organisms [12,13].

Zinc can enter the environment from industrial processes as particles released into the atmosphere, or as wastewater discharged into waterways or the ground. It is clear that human activities (heavy industry, metal industry, waste incineration, steel production and galvanization processes) can contribute to the increase in zinc concentrations within the global environment. Highly soluble zinc compounds (hydrated zinc cations) can migrate into the ground water, lakes, streams and rivers where they interact with organic and inorganic matter [12]. The toxicological properties of zinc and its compounds, the possibility of migration and the risk of environmental pollution make it necessary to remove zinc ions from polluted waters and wastewater before they are released into the environment.

Recent studies have shown that polluted waters or industrial wastewaters may contain varying amounts of zinc, for example, 52.8 mg/L (electroplating company, Atom, Dubna, Russia) [14], 10 mg/L, being approximately 80% and 20% in the form of Zn^2+^ and ZnSO_4_ (aq), respectively [15], 33.3 g/L (spent acid solution from the pickling stage of a galvanizing plant) [16], 1392.1 mg/L (zinc plating industry) [17], 22.7 mg/L (galvanic wastewater) [18] and 49.8 mg/L (zinc electroplating industry) [19].

Several treatment methods have been used to remove heavy metal cations from industrial wastewater. For the removal of zinc from wastewater, adsorption on cork powder [20], a complexation–microfiltration process [21], commercial activated carbon [22], polyaniline nanocomposite coated on rice husk [23], ferric chloride, alum and anionic polymer [24] were used. Generally, the removal of heavy metals is accomplished using a number of conventional methods including ion exchange, precipitation, coagulation, filtration, reverse osmosis and even solvent extraction [25]. Chemical precipitation is widely used in the industry and is considered as one of the cheapest, most simple and easy to implement methods. It consists of the use of alkaline reagents (NaOH, CaO, Ca(OH)_2_ and Na_2_CO_3_) for the precipitation of sparingly soluble metal hydroxides in which its solubility depends on the pH of the wastewater. However, this method has some limitations, i.e., using high doses of alkaline reagents, large volumes of sludge and limited effectiveness in the case of amphoteric hydroxides and in the presence of complexing compounds [18,26,27,28,29,30,31]. In these cases, additional precipitants are used to ensure that the concentration of metals in the wastewater treatment is reduced to the legal requirements. For example, for the removal of copper, HTDC (1,3,5-hexahydrotriazinedithiocarbamate) [32], DTC (dimethyldithiocarbamate sodium salt) [33], potassium ethyl xanthate and insoluble starch xanthate (ISX) [34,35,36] were used. The use of conventional alkaline precipitation with the sulfur compounds allows not only for reduced operating costs, but also for obtaining wastewater that contains low concentrations of heavy metals. The use of sodium trithiocarbonate (Na_2_CS_3_) may be a part of this concept. Sodium trithiocarbonate was used for wastewater containing copper, nickel and tin derived from galvanic and printed circuit boards on both laboratory and industrial scales [18,29,30,31]. Research has been carried out that uses model solutions containing heavy metals and confirmed the possibility of the effective removal of 94.0–99.9% of copper and 24.3–94.2% of nickel at pH 1–13 [37]. 

The aim of this research was the synthesis, testing of properties and the application of sodium trithiocarbonate (Na_2_CS_3_) for the precipitation of zinc from concentrated galvanic wastewater containing complexing compounds determined and recalculated as EDTA (ethylenediaminetetraacetic acid). Prior to the use of sodium thiocarbonate, conventional chemical precipitation using sodium hydroxide (NaOH), lime (Ca(OH)_2_) and soda (Na_2_CO_3_) to select the most effective reagent was applied. For the optimization of the proposed method, Central Composite Design (CCD) and response surface methodology (RSM) were applied. In addition, toxicity tests of raw and treated wastewater were carried out using the Activated Sludge Activity Test (ASAT, dehydrogenase activity) as well as to test plants such as the white mustard *Sinapis alba* and the garden cress *Lepidium sativum* L.

## 2. Results

### 2.1. Selected Physicochemical Parameters of the Solution of Sodium Trithiocarbonate Used in the Study (Na_2_CS_3_)

The selected physicochemical parameters of synthesized sodium trithiocarbonate are presented in Table 1. 

### 2.2. Selected Physicochemical and Toxicological Parameters of Concentrated Galvanic Wastewater Used in the Study

The selected physicochemical and toxicological parameters of raw galvanic wastewater used in the study are showed in Table 2.

### 2.3. Removal of Zinc from Galvanic Wastewater by Sodium Carbonate (Na_2_CO_3_), Calcium Hydroxide Suspension (Ca(OH)_2_), and Sodium Hydroxide (NaOH)

**Figure 1:**
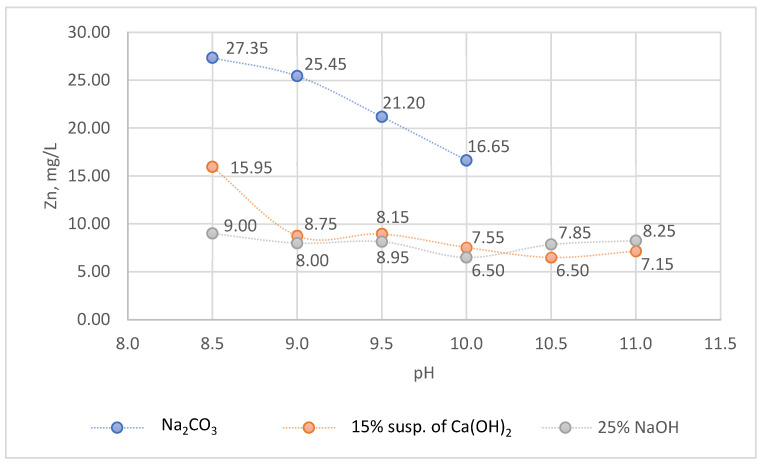
The concentration of zinc in concentrated galvanic wastewater treated by 20% solution of Na_2_CO_3_, 15% suspension of Ca(OH)_2_ and 25% solution of NaOH (the pH value of the 20% solution of Na_2_CO_3_ was 10.5 ± 0.1).

The changes in the concentration of zinc in galvanic wastewater during chemical precipitation by various alkaline reagents are depicted in Figure 1.

### 2.4. The Optimization of the Removal of Zinc (Zn) from Galvanic Wastewater by Sodium Trithiocarbonate (Na_2_CS_3_)

The experimental conditions (combinations of the input parameters) for 16 experiments are depicted in Table 3.

The analysis of the experiments’ findings before and after eliminating statistically insignificant parameters are shown in Table 4 and Table 5, respectively.

Table 6 presents the results of verification of the adequacy of the model by ANOVA and Table 7 depicts calculated linear (L) and quadratic (Q) coefficients for the developed statistical model.

**Figure 2:**
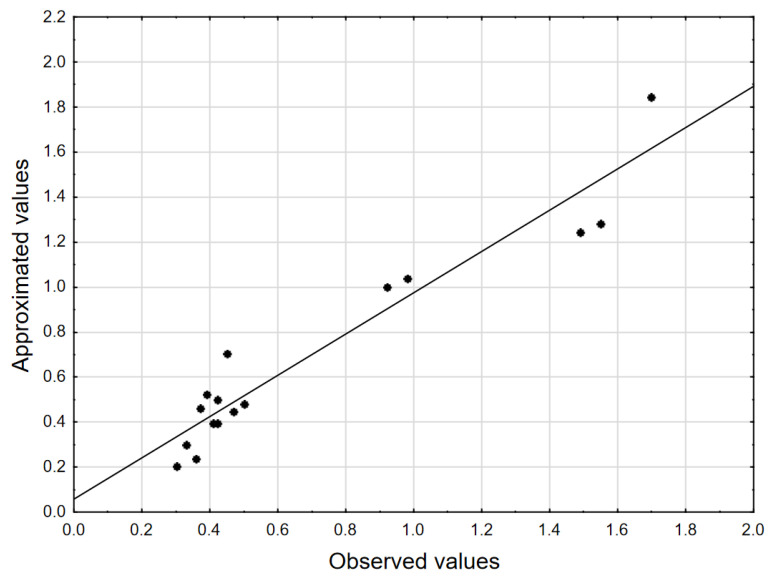
Approximated vs. observed values (Zn, mg/L, 3 value, 1 block, 16 experiments, MS = 0.0320, R^2^ = 0.9173, R^2^_adj_ = 0.8622).

**Figure 3:**
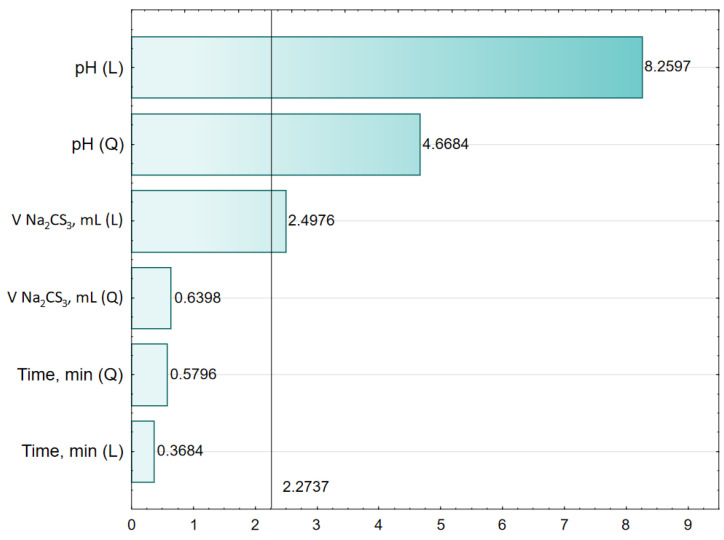
Bar chart of standardized effects (3 value, 1 block, 16 experiments, MS = 0.0320, R^2^ = 0.9173, R^2^_adj_ = 0.8622, L—linear effect, Q—quadratic effect, 2.2737—the absolute value of the standardized effect assessment for *p* = 0.05).

Figure 2 shows the relationship between the observed and estimated efficiency (Zn, mg/L) from the model. Figure 3 presents a bar chart of the standardized effects. The 2.2737 value indicates the absolute value of the standardized effect assessment for *p* = 0.05.

**Figure 4:**
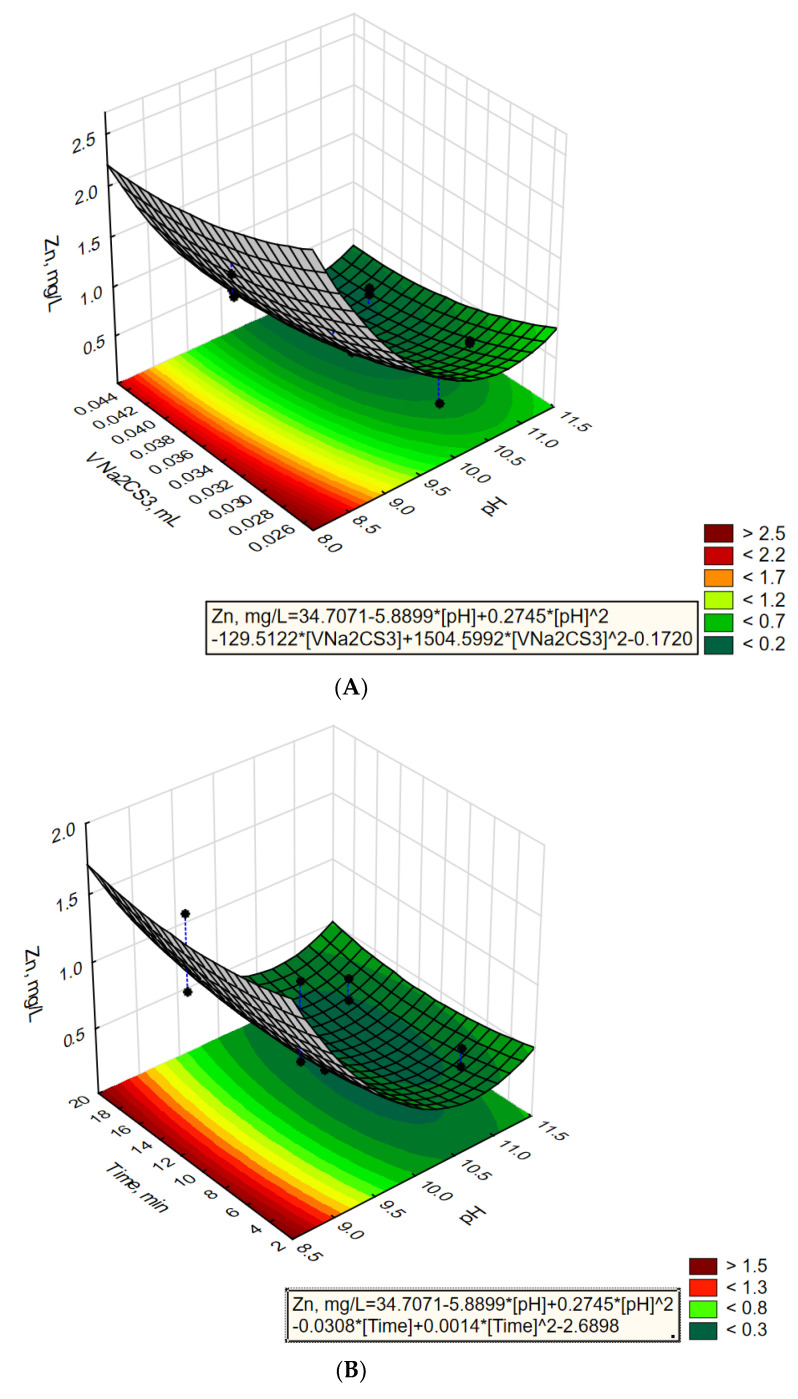

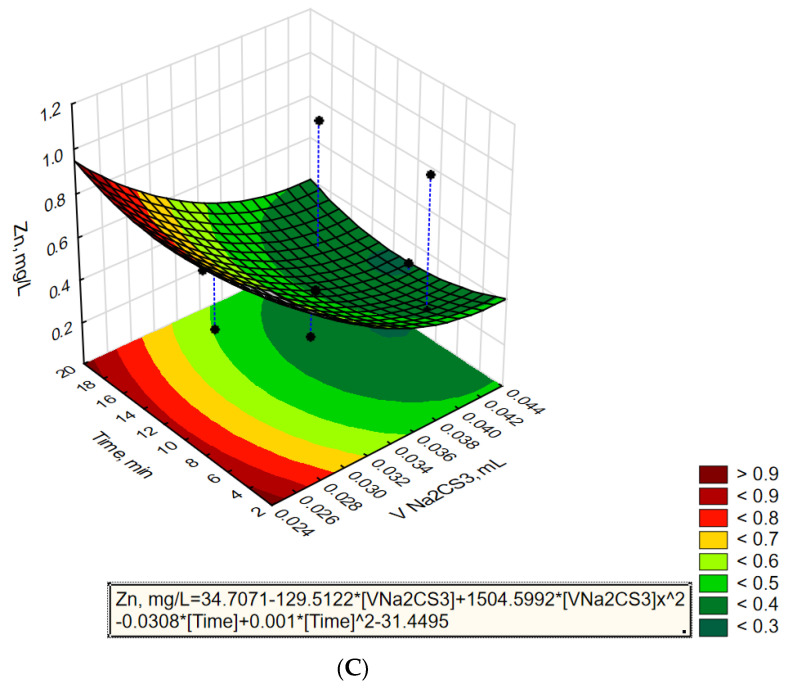
Response surface plots for Zn, mg/L with respect to pH and V Na_2_CS_3_ dose, for constant time = 10 min (**A**), pH and time (min) for constant V Na_2_CS_3_ dose = 0.035 mL/L (**B**), and V Na_2_CS_3_ dose and time (min) for constant pH = 10 (**C**).

Figure 4a–c show the changes in the value of the output parameter (Zn, mg/L) depending on the combination of two independent parameters i.e., pH value, V Na_2_CS_3_ dose, mL, and time, min. The value of the third independent parameter not shown in the graph is immutable (i.e., time, min for **4a** V Na_2_CS_3_ dose, mL for **4b** and pH value for **4c**).

Table 8 presents the findings of the experimental model verification developed for the galvanic wastewater treatment using the combination of Ca(OH)_2_ and Na_2_CS_3_ in optimal conditions. Table 9 shows the changes in the selected physicochemical parameters of treated wastewater after treatment under optimal conditions calculated from the model.

### 2.5. The Assessment of the Phytotoxicity of Galvanic Wastewater before and after Treatment by Sodium Trithiocarbonate (Na_2_CS_3_)

**Figure 5:**
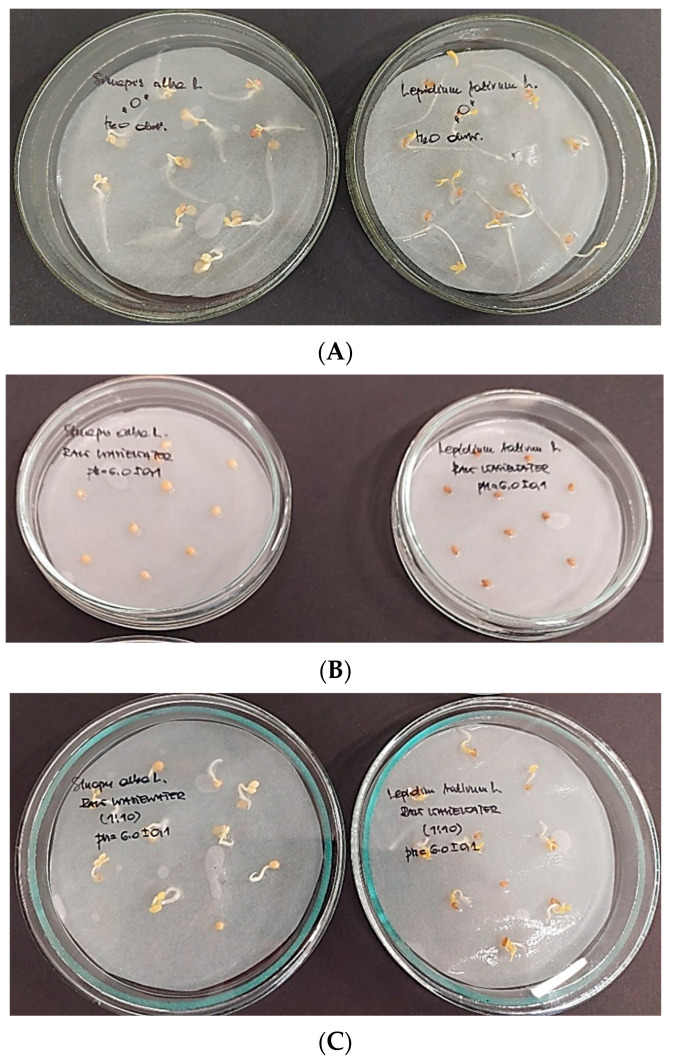

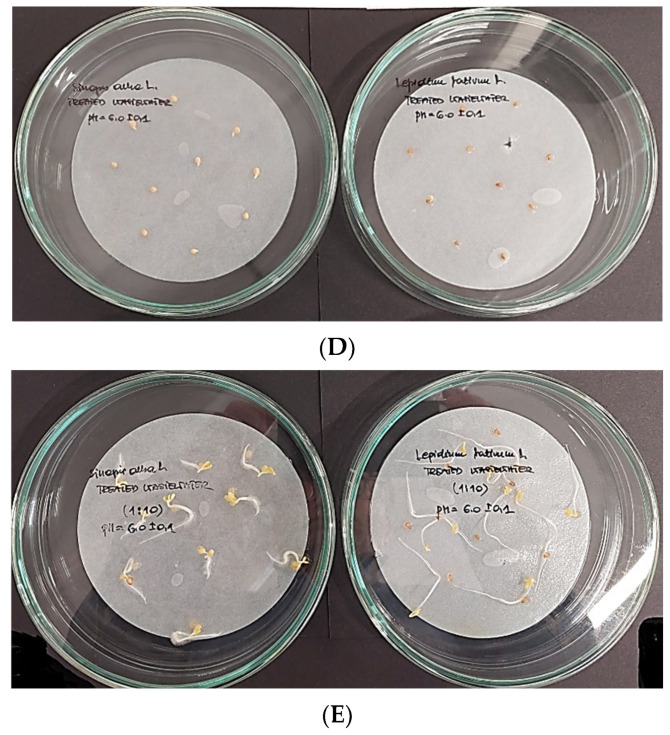
Phytotoxicity test results for *S. alba* and *L. sativum* L. (from left to right); blank—distilled water (**A**), raw wastewater, pH 6.0 ± 0.1 (**B**), raw wastewater (1:10), pH 6.0 ± 0.1 (**C**), treated wastewater pH 6.0 ± 0.1 (**D**), treated wastewater (1:10) pH 6.0 ± 0.1 (**E**).

Figure 5 shows the results of phytotoxicity tests conducted with the white mustard (*Sinapis alba*) and the garden cress (*Lepidium sativum* L.) applied as test organisms.

**Figure 6:**
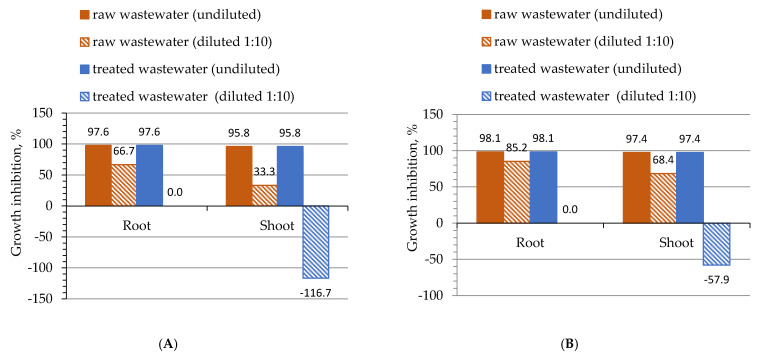
Phytotoxicity test findings; growth inhibition of roots and shoots on the raw and treated (in optimal conditions) real galvanic wastewater samples (**A**) *S. alba*, (**B**) *L. sativum* L.

**Figure 7:**
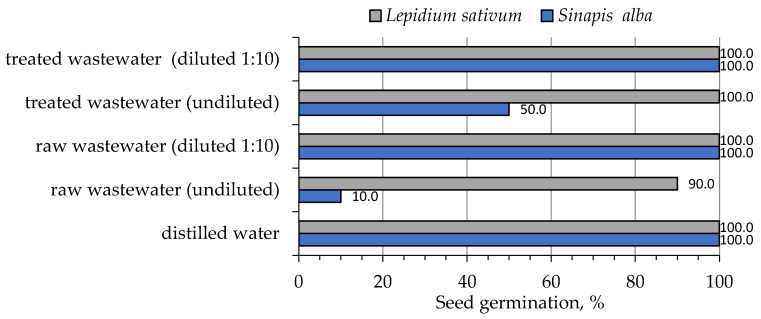
Seed germination of *S. alba* and *L. sativum* L. for undiluted and diluted (1:10) wastewater samples.

Figure 6 shows the results of the percentage of growth inhibition (GI) for undiluted and diluted (1:10) raw and treated galvanic wastewater. Figure 7 shows seed germination (%) of both tested plants and the results of the one-way ANOVA test for the phytotoxicity test performed (Table 10).

Table 10 shows that the average length of roots and shoots between the groups were significantly different (*p* < 0.05). 

**Figure 8:**
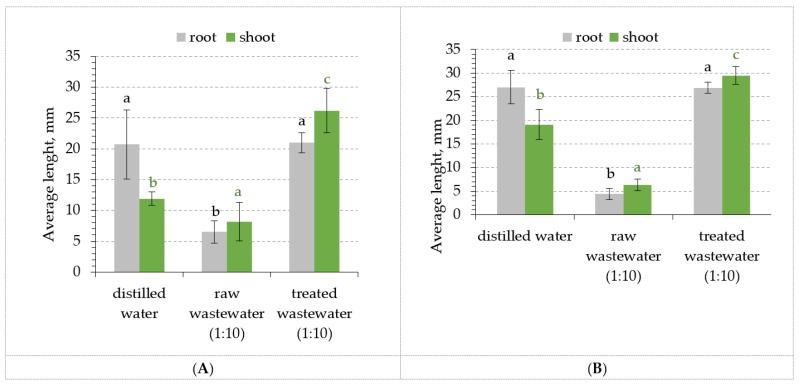
The lengths of roots and shoots of tested plants for distilled water (blank sample), raw and treated wastewater (1:10); (**A**) *S. alba*, (**B**) *L. sativum* L. The combinations of different letters indicate statistically significant differences at *p* < 0.05 (one-way ANOVA, post hoc Tukey HSD test).

In order to clarify which groups differ from each other the post hoc Tukey Honestly Significant Difference (Tukey HSD) analysis was carried out. The obtained findings are presented in Figure 8.

## 3. Discussion

### 3.1. Selected Physicochemical Parameters of the Solution of Sodium Trithiocarbonate Used in the Study (Na_2_CS_3_)

As a result of the synthesis, after phase separation and filtration of the product, a clear, transparent, dark red and alkaline solution of Na_2_CS_3_ with a characteristic odor was obtained. The concentrations of post-reaction impurities (SO_3_^2−^ + S_2_O_3_^2−^, 0.20 ± 0.05%) were low compared to the concentration of the finished product (Na_2_CS_3_, 39.5 ± 0.1%). The Na_2_CS_3_ solution with similar quality parameters using the same synthesis conditions was obtained previously [18]. In the obtained solution the concentration of heavy metals as impurities was determined. Among the determined cations, the highest concentration was iron. In order to determine the elemental composition of the obtained product, it was subjected to lyophilization and then CHNS analysis was performed. After the lyophilization process, a yellow amorphous powder was obtained. The preparation of yellow Na_2_CS_3_·2H_2_O crystals has been described in the previous literature [38]. Based on the hydrogen content in the tested sample, it can be concluded that the chemical composition of the obtained product corresponds to the formula of Na_2_CS_3_·H_2_O. The theoretical carbon and sulfur content of Na_2_CS_3_·H_2_O is 6.97% and 55.87%, respectively. Similar values were obtained for the tested product, i.e., 6.73% and 49.01%. It should be taken into account that the uncertainty of the CHNS analysis is approximately 10%.

### 3.2. Selected Physicochemical and Toxicological Parameters of Concentrated Galvanic Wastewater Used in the Study

Physicochemical tests of raw galvanic wastewater showed that it contained not only significant amounts of zinc (1534 ± 77 mg/L, concentrations of other metals were many times lower), but also organic compounds determined as COD and TOC (2900 ± 145 mg O_2_/L and 985 ± 49 mg/L, respectively). The concentration of total nitrogen indicated that some of the organic compounds present in wastewater were in the form of nitrogenous compounds. In addition, it was shown that wastewater contained complexing agents determined as EDTA (Table 2). The consequence of the above wastewater composition was the complete inhibition of the activated sludge dehydrogenase activity (Table 2) and the negative effects on germination, root and shoot growth in test plants i.e., *S. alba* and *L. sativum* L. (Figure 6, Figure 7 and Figure 8). This indicated a significantly high toxicity of wastewater in relation to activated sludge micro(organisms) and test plants.

Studies conducted by other authors indicate that the composition and physicochemical parameters of (spent) wastewater from galvanizing processes may vary significantly. For example, a spent zinc plating bath from a metal finishing contained 200 mg Zn, and pH, TSS, COD and oil–grease were 2.6, 142 mg/L, 600 mg/L and 44 mg/L, respectively [39]. Other studies have shown the following composition of galvanic Zn-bearing wastewater: Zn (81 mg/L), Fe (1.4 mg/L) and Al (0.6 mg/L), and it had a pH value of 4.8. Nitrate, phosphate and COD were 3.6 mg/L, 10.3 mg/L and 441.2 mg/L, respectively [40]. The findings of other research conducted for galvanic wastewater revealed that pH and COD were 0.7 and 4000 mg O_2_/L, respectively. In this case, the concentrations of Cr, Cu, Zn, Hg, Pb and Co were 3956, 1499, 972, 65, 26 and 3.2 mg/L, respectively [41].

Within the context of heavy metals and their negative impacts, including zinc, upon the activity of activated sludge dehydrogenase, it was found that zinc sulfate (and actually zinc cations) inhibits the activity of this enzyme [42]. The literature data confirm that an effect of zinc on the dehydrogenase activity of *Escherichia* sp., *Proteus* sp. and *Pseudomonas* sp. isolated from river water depends on zinc concentrations. The dehydrogenase activity on the above species was progressively inhibited with the increase in zinc concentrations within the water [43]. It has been proven that Zn is a trace element that is necessary for many biochemical processes in living organisms; however, in concentrations higher than required, it is a strong inhibitor of respiratory activity in microorganisms. It seems most likely that the inhibition of dehydrogenase activity is similar to the non-competitive inhibition of enzymes, where the competitive inhibitor binds to an area of the enzyme other than the active site and the allosteric site. Zinc cations are not analogues of the substrate and cannot competitively bind to the active site of dehydrogenase enzymes [43,44,45,46,47]. This study supports that the effect of zinc on the germination and growth of the roots and shoots of *L. sativum* depends on the form and concentration of the metal, and, consequently, the degree to which it activates the plant’s antioxidant enzymatic system [48].

### 3.3. Removal of Zinc from Galvanic Wastewater by Sodium Carbonate (Na_2_CO_3_), Calcium Hydroxide Suspension (Ca(OH)_2_) and Sodium Hydroxide (NaOH)

Alkaline reagents, i.e., Na_2_CO_3_, Ca(OH)_2_ and NaOH were used for the initial precipitation of zinc ions (Figure 1). A significant reduction in the concentration of Zn ions in the case of Na_2_CO_3_ use was noted at pH 10 (16.65 mg/L). In the case of Ca(OH)_2_ and NaOH, the concentrations of Zn ions in treated wastewater decreased at pH 9 and were 8.75 and 8.00 mg/L, respectively. As the pH of the effluent increased, the concentration of Zn ions remained in the range of 6.50–8.95 mg/L. Ca(OH)_2_ was more effective than Na_2_CO_3_ and NaOH (Figure 1), and therefore, subsequent experiments with the use of sequential precipitation (Ca(OH)_2_ + Na_2_CS_3_) were carried out using Ca(OH)_2_. When Ca(OH)_2_ and NaOH are used, zinc hydroxide is precipitated, which, depending on the pH of the wastewater, can turn into Zn^2+^-ions or [Zn(OH)_4_] ^2−^ according to the reaction (1):

Zn^2+^ ⇄ Zn(OH)_2_ ↓ ⇄ [Zn(OH)_4_] ^2−^ (1)

In the case of soda in the pH range of 7–9, more than 90% of the precipitate is basic zinc carbonate (2ZnCO_3_·3Zn(OH)_2_) [49]. Due to the amphoteric properties of zinc, the precipitated hydroxide can be dissolved, which results in an increase in the concentration of zinc within the treated wastewater. This phenomenon is unfavorable from the point of view of wastewater treatment technology. Another problem is the presence of complexing compounds (e.g., EDTA) that prevent the precipitation of zinc after adding alkaline reagents and increasing the pH value. As the conducted research showed, the above-mentioned limitations of the applied wastewater treatment method can be eliminated by sequential precipitation using Ca(OH)_2_ and Na_2_CS_3_.

### 3.4. Optimization of the Removal of Zinc from Galvanic Wastewater by Sodium Trithiocarbonate (Na_2_CS_3_)

Optimizing the process of zinc precipitation from galvanic wastewater was carried out with the use of CCD and RSM (Table 3). The highest efficiency of the purification process was observed in experiment eight. In this case for pH 11, Na_2_CS_3_ dose 0.04 mL/L and time 15 min, the concentration of Zn in treated water was 0.30 ± 0.03 mg/L. It suggests that the purification process is probably more effective with an alkaline reaction, a higher dose of Na_2_CS_3_ and a short time period. As a result of the reaction of Na_2_CS_3_ with zinc ions, ZnCS_3_ is precipitated in the form of a sparingly soluble precipitate, according to the reaction (2):

Zn^2+^ + CS_3_^2−^ → ZnCS_3_↓ (2)

The precipitation of other metals present in small amounts in the treated wastewater proceeded according to analogous reactions as for zinc. The literature data [50,51,52,53] confirm the possibility of forming the metals’ trithiocarbonates according to the reactions (3)–(7):

Cu^2+^ + CS_3_^2−^ → CuCS_3_↓ (3)

Ni^2+^ + CS_3_^2−^ → NiCS_3_↓ (4)

Cd^2+^ + CS_3_^2−^→ CdCS_3_↓ (5)

Pb^2+^ + CS_3_^2−^ → PbCS_3_↓ (6)

Fe^2+^ + CS_3_^2−^ → FeCS_3_↓ (7)

The statistical analysis of the results, presented in Table 3, showed (Table 4 and Table 5) that some independent factors (linear interactions) are statistically insignificant and can be removed from the model. After that R^2^ and R^2^_adj_ are calculated as follows in (8) and (9):

(8) $$R^{2}=1-\frac{SS_{residual}}{SS_{model\;}+SS_{residual}}$$

(9) $${R^{2}}_{\mathrm{adj}}=1-\frac{n-1}{n-p}\;(1-R^{2})\;$$

where SS is the sum of the squares, n is the number of experiments, p is the number of predictors, not counting the constant values that were changed from 0.9421 to 0.9173 (for R^2^) and from 0.8552 to 0.8622 (for R^2^_adj_). It is clear that adding more statistically insignificant input parameters to the model causes a decrease in the R^2^_adj_ value, whilst adding more significant parameters causes an increase in the R^2^_adj_ value and generally R^2^ ≥ R^2^_adj_. Other studies indicate that R^2^ and R^2^_adj_ can take similar values, e.g., R^2^ = 0.8799 and R^2^_adj_ = 0.7998 for the coagulation process for river water containing pharmaceuticals [54], R^2^ = 0.9461 and R^2^_adj_ = 0.7379 for the textile wastewater treatment process by Fenton reaction [55], and R^2^ = 0.9957 and R^2^_adj_ = 0.9937 for the sonophotocatalytic treatment of AB113 dye and real textile wastewater using ZnO/persulfate [56]. On the one hand, the high value of the coefficient of determination (R^2^ = 0.9173) suggests that the model cannot explain 0.0827 of the total variation, whereas, on the other hand, the high value of the adjusted coefficient of determination indicates the significance of the model parameters (R^2^_adj_ = 0.8622). R^2^ = 0.9173 allows a conclusion to be made about a good fit of the data estimated from the model to the experimental data. Generally, it can be assumed that if 0.8 < R^2^ ≤ 0.9, the fit of the model is good [54]. 

The adequacy of the model coefficients was verified by means of ANOVA (Table 6). The calculated intercept, linear (L) and quadratic (Q) coefficients of the fitted model are presented in Table 7. The regression model enables the modeling of the response as a mathematical function of several continuous factors, and good estimates of the model parameters are necessary. Each response (e.g., Zn, mg/L) can be expressed by a mathematical equation that describes the response surface. The following second order polynomial equation is suitable for a mathematical description of the response function (10):

(10) $$\mathrm{Efficiency},\%=\beta_{0}+\sum_{i=1}^{k}\beta_{i}x_{i}+\sum_{i=1}^{k}\sum_{j=i+1}^{k}\beta_{\mathrm{ij}}x_{i}x_{j}+{\;\beta }_{1}x_{1}+\sum_{i=1}^{k}\beta_{\mathrm{ii}}x_{i}^{2}$$

where Efficiency, % is the dependent parameter, β_0_ is the constant coefficient, β_i,_ β_ij_ and β_ii_ are the coefficients of linear and quadratic interactions, respectively, k is the number of independent parameters and x_i_ are the input predictors or controlling variables (i = 1, 2) [56]. Therefore, the concentration of Zn, mg/L, can be expressed as follows (11) (numerical values are rounded to three decimal places):

Zn, mg/L = 34.707 − 5.890 [pH] + 0.274 [pH]^2^ − 129.512 [V Na_2_CS_3_] + 1504.599 [V Na_2_CS_3_]^2^ − 0.031 [Time] + 0.001 [Time]^2^ (11)

Figure 2 shows a graph of approximated values relative to the observed values. In the case where there is a good fit of the model to the experimental data, the measurement points are close to the straight line. Figure 3 shows a bar chart of standardized effects. It can be seen that the greatest impact on the value of the dependent parameter (Zn, mg/L) has three independent factors, i.e., pH(L), pH(Q) and V Na_2_CS_3_(L). The next three factors, i.e., V Na_2_CS_3_(Q), Time(L) and Time(Q) are not statistically significant.

Figure 4a–c show response surface plots for zinc concentrations in treated wastewater (Zn, mg/L). From the plot of the response surface in Figure 4b,c, it can be seen that the reaction time is a statistically insignificant parameter, which is confirmed by previous calculations (Table 7). On the one hand, the precipitation reaction occurs quickly as it occurs immediately after the addition of sodium trithiocarbonate. On the other hand, it should be taken into account that the highest efficiency of the process can be obtained at a pH of about 10.75 and with a dose of sodium trithiocarbonate of 0.043 mL/L.

For the most favorable conditions adopted from the model (pH = 10.75 ± 0.10, V Na_2_CS_3_ dose = 0.043 mL/L, and Time = 5 min), an experimental verification of the model was carried out. In addition, the change in zinc concentration in treated wastewater over time, i.e., after 5, 10, 15 and 20 min, was examined (Table 8). The obtained test results showed that the concentration of zinc did not change over time, which was in line with the predictions of the model. In addition, the concentration of zinc in the treated wastewater after a 5 min reaction time was comparable to the concentration calculated from the model (0.21 vs. 0.15 mg/L).

The purification of galvanic wastewater in the most favorable conditions determined from the model resulted in a decrease in the zinc concentration by 99.99%, i.e., from 1534 ± 77 mg/L to 0.15 ± 0.03 mg/L (Table 9). The concentrations of nickel and iron were 0.074 ± 0.001 mg/L and 0.115 ± 0.003 mg/L, respectively. The concentrations of other metals were less than 0.05 mg/L. Based on published data, the removal rate of zinc by Na_2_CS_3_ was 56.3–91.7 (for raw wastewater), 48.8–87.2 (for a mixture of raw industrial wastewater and rain water), 97.0–99.0% (for raw galvanic wastewater) and >99.9% (for artificial galvanic wastewater [52]. In other studies, >99.9% efficiency (Zn) for artificial wastewater containing Zn, Fe, Cu, Cr, Pb, Cd and Ni has been achieved [53]. For the precipitation–flotation process, 99% efficiency was obtained for the mixture solution of Fe(III), Zn and Cd at pH 10.3, after 15 min of treatment [57]. Other methods have also been used to remove zinc from wastewater. For the copper and zinc removal from wastewater, alum sludge recovered from a water treatment plant was applied. The findings demonstrated that the high removal efficiency (97.4% and 96.6% for zinc and copper, respectively) was obtained at pH 6 using a high amount of sludge (1.4 g) [58]. Other studies indicated that the application of an electrocoagulation process for high strength industrial electroplating wastewater (pH 2, COD 1 430 mg O_2_/L, Ni 150 mg/L, Cu 30 mg/L, Zn 25 mg/L and Fe 2.9 mg/L) made it possible to remove 92.1% Ni, 87.8% Zn and 82.9% Cu [59]. In the case of using agricultural wastes as adsorbents, the highest efficiency of zinc removal (63.6%) was obtained at pH 6 [60]. Based on the literature data, the presented method of using sodium trithiocarbonate for wastewater treatment is characterized by high efficiency.

Our research has shown that in addition to removing metals from wastewater, the Activated Sludge Activity Test (ASAT) indicates measurable dehydrogenase activity values (−70% for undiluted and −15% for diluted wastewater). The issues related to the occurrence of heavy metal cations in municipal wastewater concern not only potential problems related to wastewater treatment, but also for the distribution of heavy metals in the treatment system as well as for their presence in the sludge and its agricultural use [61].

### 3.5. Toxicological Findings

Figure 5 shows the results of phytotoxicity tests that were conducted on white mustard (*S. alba*) and garden cress (*L. sativum* L.), which were used as test plants. The lowest GI and growth stimulation effect was observed for treated wastewater after its dilution (−116.7 for *S. alba* shoot and −57.9 for *L. sativum* L. shoot). Dilution of raw wastewater (Figure 6) allowed the GI of *S. alba* to decrease to 66.7% (roots) and 33.3% (shoot) and 85.2% and 68.4% for *L. sativum* L. roots and shoots, respectively. It was noticed that the calculated GI for wastewater samples without dilution was comparable and ranged between 95.8 and 97.6%.

The germination of *S. alba* (Figure 7) was recorded as 100% for all samples except raw undiluted wastewater (90%). The germination of *L. sativum* L. was 100% for both diluted wastewater and distilled water (blank sample). However, for undiluted wastewater, the values of germination were only 10% and 50% (*S. alba*).

The results of the phytotoxicity tests revealed that the average length of *S. alba* sprouts on treated wastewater samples (diluted 1:10) reached 21.0 mm for roots (in blank sample 20.7 mm). However, it was observed that the length of shoots for treated wastewater reached 26.2 mm and was higher than the length of shoots for distilled water (11.9 mm). The estimated length of roots and shoots for raw wastewater (diluted 1:10) were only 4.4 mm and 6.3 mm, respectively. Furthermore, the phytotoxicity test for *L. sativum* L. showed equally low values for raw wastewater samples, i.e., 4.4 mm for roots and 6.3 mm for shoots, in comparison with treated wastewater, 26.9 mm and 29.5 mm, respectively. ANOVA indicated (Table 10) that the treatment process had a significant effect on plant growth. The results of the Tukey HSD multiple comparison test (Figure 8) showed that the lengths of the roots and shoots for *S. alba* and *L. sativum* L. were significantly different between samples (*p* < 0.001). The better results of phytotoxicity tests may have been influenced by the low content of Zn in treated water. 

It is clear that the trace amounts of Zn are important cations of various enzymes, e.g., dehydrogenase [62]. However, the excess content of Zn in soil, contaminated by industrial wastewaters, can reduce seed germination and lead to toxic symptoms in plants such as chlorosis or necrosis [63,64]. The inhibitory impact of zinc and other heavy metals (e.g., Cd, Cu, Fe, Pb) on *S. alba* growth was also studied by other researchers. The study showed that heavy metals can reduce chlorophyll content, but, simultaneously, the concentration of Zn in roots and shoots is the lowest. The interpretation of our research seems to indicate that high concentrations of Zn in raw wastewater was not the only factor for the lack of germination of the test organisms [65].

## 4. Materials and Methods

### 4.1. Reagents, Chemicals and Synthesis of Na_2_CS_3_

For research, all chemicals except those used for synthesis of Na_2_CS_3_ were at least analytical grade. In addition, deionized water (<0.08 µS/cm) was used for the preparation of chemicals and dilution of wastewater samples. The synthesis of Na_2_CS_3_ was performed as previously described [18]. The solution of Na_2_CS_3_ was lyophilized before CHNS analysis. For pH adjustment (precipitation of heavy metals), 20% solution of Na_2_CO_3_, 15% suspension of Ca(OH)_2_, 25% solution of NaOH and 5% solution of H_2_SO_4_ were used (Chempur, Piekary Śląskie, Poland). For determination of heavy metals by FAAS, ultrapure concentrated nitric acid (HNO_3_) and hydrochloric acid (HCl) were used (sample digestion in *aqua regia*).

### 4.2. Origin of the Galvanic Wastewater and Sampling Methodology

Wastewater samples came from a galvanizing plant located in the south of Poland. Sampling was performed manually and poured into two-liter polypropylene bottles in accordance with PN-EN ISO 5667-6:2016-12 [66], every hour for a 12 h period. The two-liter samples were thoroughly mixed and an average sample was obtained. The average sample was transported in a polypropylene container to the laboratory under refrigeration conditions (8 °C) and stored in a refrigerator (4 °C) until the tests were performed. The sample was not fixed.

### 4.3. Analytical Methods

The determinations of pH value, specific electrical conductivity (SEC) and salinity were performed electrometrically (CPC-401, Elmetron, Zabrze, Poland) according to PN-EN ISO 10523:2012 [67] and PN-EN 27888:1999 [68], respectively. Turbidity and color were performed using spectrophotometer (PF-11, Macherey-Nagel GmbH, Düren, Germany) in accordance with the guidelines described in PN-EN ISO 7027-1:2016-09 and PN-EN ISO 7887:2012, respectively [69,70]. Gravimetric methods described in PN-EN 872:2007 and PN-ISO 9280:2002 were used for the evaluation of Total Suspended Solids (TSS) and sulfate, respectively [71,72]. The concentration of chloride was determined titrimetrically by Mohr’s method according to PN-ISO 9297:1994 [73]. Chemical Oxygen Demand (COD), Total Organic Carbon (TOC), Total P (TP) and total nitrogen (TN) were determined spectrophotometrically (PF-11 and NANOCOLOR 500D spectrophotometer) by test kits (Macherey-Nagel, Düren, Germany). Complexing agents (calculated as EDTA) were determined spectrophotometrically by test kits (PF-11 spectrophotometer Macherey-Nagel, Düren, Germany). Heavy metals (Zn, Cu, Ni, Cd, Pb, Fe) were determined by FAAS (Solaar S4, Thermo Fisher Scientific, Waltham, MA, USA) according to PN-ISO 8288:2002 with prior digestion with *aqua regia*. [74]. Activated Sludge Activity Tests (ASAT) were performed by test kit Nanocolor TTC/Sludge with spectrophotometric measurement (NANOCOLOR 500D, Macherey-Nagel, Düren, Germany). Activity phytotoxicity tests were performed according to EN ISO 18763 using *S. alba* and *L. sativum* L. as test organisms for undiluted and diluted (1:10) wastewater (in each case, pH of the wastewater was adjusted to 6.0 ± 0.1). A total of 5.0 ± 0.1 mL distilled water (blank sample) or wastewater was placed in 9 cm Petri dishes and lined with filter paper of 87 g/m^2^ (grade 292, Munktell, Ahlstrom Munksjö, Finland). Then, ten seeds of the tested plant were placed out on moist filter paper and closed with the lid. To determine the effect on germination and growth, the seeds were incubated in darkness at 25 ± 1 °C for 72 h. The lengths of roots and shoots were measured with accuracy ±0.1 cm. Each experiment was conducted in triplicate. The values of the inhibition of roots and shoots (GI, growth inhibition) were calculated according to Equation (12):

(12) $$\mathrm{GI}=\frac{A-B}{A}\cdot 100\%$$

where A—an average length of roots/shoots (blank sample), B—an average length of roots/shoots (test sample) [75].

The selected physicochemical parameters of synthesized Na_2_CS_3_ (concentration of Na_2_CS_3_, concentration of SO_3_^2−^ and S_2_O_3_^2−^, density, substances insoluble in H_2_O) were determined according to PN-C-84042:1977 [76]. The concentration of Na_2_CS_3_ was recalculated as follows:

% of Na_2_CS_3_ = % of Na_2_S × 1.976 (13)

(molar ratio of 1 mole of Na_2_CS_3_ to 1 mole of Na_2_S is 154.89 g/mol:78.05 g/mol = 1.976).

### 4.4. Design of Experiments (Central Composite Design, CCD, and Response Surface Methodology, RSM)

In order to optimize the Zn removal from concentrated galvanic wastewater, CCD/RSM was used for three independent parameters, i.e., pH, V Na_2_CS_3_ dose (mL/L) and time (min). On the basis of preliminary experiments (effectiveness of conventional treatment by 20% solution of Na_2_CO_3_, 15% suspension of Ca(OH)_2_ and 25% solution of NaOH) and literature data, the ranges of values of independent parameters were adopted, i.e., pH value (9.0–11.0, according to Figure 1), V Na_2_CS_3_ dose (mL/L) (0.03–0.04 mL/L; stoichiometric doses of 39.5% solution of Na_2_CS_3_ for 8.75 mg/L Zn (pH = 9, after Ca(OH)_2_ treatment), 7.55 mg/L Zn (pH = 10, after Ca(OH)_2_ treatment) and 7.15 mg/L Zn (pH = 11, after Ca(OH)_2_ treatment) and were 0.038, 0.033 and 0.031 mL/L. The concentrations of Zn in treated wastewater after applying 15% suspension of Ca(OH)_2_ and 25% solution of NaOH were comparable; however in the case of lime, the sludge flocs were larger and more quickly sedimented than in the case of NaOH. Therefore, the pretreatment of the wastewater was carried out with lime. In initial tests, the precipitation of zinc was rapid; therefore, the reaction time of 5 to 15 min was adopted. The planning of the experiments was performed using CCD and Statistica 13 (TIBCO Software Inc., Palo Alto, CA, USA). The application of CCD made it possible to obtain the experimental plan presented in Table 3. The experimental plan consisted of 16 experiments with two experiments at the center of the plan (15C and 16C), and was the combination of the initial values of three independent parameters (pH, V Na_2_CS_3_, mL/L and time, min). On the basis of the Zn concentration before and after precipitating process, the Zn removal efficiency (%) was calculated.

Additionally, the following parameters were assumed to be constant: temperature (20 ± 1 °C), stirring speed (500 RPM) and volume of the treated water (500 mL in each experiment).

### 4.5. Experimental Study

The experiments were performed as the jar tests with the use of 600 mL beakers and magnetic stirrers with adjustable mixing speed (Magnetic Stirrer 06-MS-PB, Chemland, Poland). In each experiment, 500 mL of raw galvanic wastewater was measured into the beaker, the appropriate volume of 15% suspension of Ca(OH)_2_ was added to achieve the fixed pH value, shown in Table 3. After that, the appropriate volume of 39.5% solution of Na_2_CS_3_ was added. The precipitation of Zn was conducted for a specific period of time (Table 3). After a set time, the agitation was turned off and the samples were left for 15 min for the sedimentation of the precipitated sludge. The determination of turbidity and TSS were performed using unfiltered samples. In the case of other parameters, the treated wastewater was filtered through a 0.45 μm nylon syringe filter (Macherey-Nagel GmbH & Co. KG, Düren, Germany).

The obtained results of the 16 experiments were analyzed using Statistica 13 to determine how the independent parameters influence the changes in the dependent parameter (Zn, mg/L). The results were evaluated statistically and dependencies between parameters were depicted and presented in 3D graphs. Experimental verification of the model was also performed in order to check whether the values of the dependent variable estimated from the model are consistent with the experimental values. All experiments were performed in triplicate.

## 5. Conclusions

The conducted research showed that the removal of zinc from concentrated galvanic wastewater by the application of conventional treatment, i.e., chemical precipitation by Na_2_CO_3_, NaOH or Ca(OH)_2_, was inefficient. The reason for this is that complexing agents are present in galvanic wastewater, which prevent the complete precipitation of heavy metals with the use of alkaline reagents. However, the use of additional precipitants such as Na_2_CS_3_ results in the formation of sparingly soluble zinc compounds (zinc sulfide and/or zinc trithiocarbonate), which consequently results in a low zinc concentration within the effluent compared to conventional methods. The presented research showed that the use of sequential precipitation (alkalization first) under the most favorable conditions (pH = 10.75 ± 0.10, V Na_2_CS_3_ dose 0.043 mL/L and time = 5 min) results in the almost complete removal of zinc from wastewater. The effectiveness of Na_2_CS_3_ is not limited by the amphoteric nature of the metal cations. Our research showed that the removal of zinc (and other impurities) led to a reduction in the inhibition of activated sludge dehydrogenase from total inhibition (for raw wastewater) to −70% (for treated wastewater). Phytotoxicity tests revealed that the seed germination index for the raw and treated wastewater increased from 10% to 50% and from 90% to 100% for white mustard (*S. alba*) and garden cress (*L. sativum* L.), respectively. Zinc removal was also associated with a statistically significant improvement in root and shoot growth. As a consequence, treated wastewater (1:10) showed a stimulating effect on plant growth and development compared to the control sample. The treated wastewater probably contained easily assimilable nitrogen or/and carbon compounds. Thus, the use of Na_2_CS_3_ is an option for the treatment of concentrated galvanic wastewater containing zinc and the process can be simply scaled up for industrial purposes.

## Figures and Tables

**Table 1:** Selected physicochemical parameters of the solution of sodium trithiocarbonate (Na_2_CS_3_).

Parameter	Unit	Result *
pH	-	13.5 ± 0.1
Appearance	-	Clear, dark red 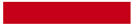
Density, at 23 °C	g/mL	1.377 ± 0.017
Concentration (Na_2_CS_3_)	%	39.5 ± 0.1
Concentration (SO_3_^2−^ + S_2_O_3_^2−^)	%	0.20 ± 0.05
Substances insoluble in H_2_O	mg/L	<0.01
Copper (Cu)	mg/L	<5
Nickel (Ni)	mg/L	<10
Zinc (Zn)	mg/L	<2
Cadmium (Cd)	mg/L	<2
Lead (Pb)	mg/L	<20
Iron (Fe)	mg/L	38.8 ± 0.2
Total carbon **	%	6.73 ± 0.24
Total hydrogen **	%	1.40 ± 0.54
Total nitrogen **	%	<0.01
Total sulphur **	%	49.01 ± 0.75

* parameter value ± standard deviation, if applicable, ** for the solid (lyophilized) Na_2_CS_3_·H_2_O.

**Table 2:** Selected physicochemical and toxicological parameters of concentrated galvanic wastewater.

Parameter	Unit	Result *
pH	-	3.1 ± 0.1
Specific electrical conductivity (SEC)	mS/cm	20.31 ± 2.03
Salinity	g NaCl/L	10.16 ± 1.02
Turbidity	FAU	19 ± 2
Color	mg Pt/L	<10
Total Suspended Solids (TSS)	mg/L	12.5 ± 1.2
Chemical Oxygen Demand (COD)	mg O_2_/L	2900 ± 145
Total Organic Carbon (TOC)	mg/L	985 ± 49
Chloride	mg/L	6300 ± 315
Sulphate	mg/L	65 ± 3
Total phosphorus (Total P)	mg/L	<0.3
Total nitrogen (Total N)	mg/L	37 ± 2
Complexing compounds (recalculated as EDTA)	mg/L	70 ± 4
Zinc (Zn)	mg/L	1534 ± 77
Copper (Cu)	mg/L	<0.05
Nickel (Ni)	mg/L	0.23 ± 0.01
Cadmium (Cd)	mg/L	<0.05
Lead (Pb)	mg/L	<0.05
Iron (Fe)	mg/L	1.01 ± 0.05
(undiluted) ASAT ** (dehydrogenase activity)	%	Complete inhibition of dehydrogenase activity
(1:10) ASAT ** (dehydrogenase activity)	%	Complete inhibition of dehydrogenase activity

* parameter value ± the measurement uncertainty for an extension factor k = 2, if applicable, ** Activated Sludge Activity Test.

**Table 3:** The experimental conditions for the CCD/RSM and results (Zn, mg/L) for the treated galvanic wastewater.

Run	Experimental Conditions			Experimental Results *
	pH	V Na_2_CS_3_ (mL/L)	Time (min)	Zn (mg/L)
1	9.0	0.030	5.0	1.55 ± 0.2
2	9.0	0.030	15.0	1.49 ± 0.15
3	9.0	0.040	5.0	0.98 ± 0.10
4	9.0	0.040	15.0	0.92 ± 0.10
5	11.0	0.030	5.0	0.50 ± 0.05
6	11.0	0.030	15.0	0.47 ± 0.05
7	11.0	0.040	5.0	0.36 ± 0.04
8	11.0	0.040	15.0	0.30 ± 0.03
9	8.3	0.035	10.0	1.70 ± 0.17
10	11.7	0.035	10.0	0.42 ± 0.04
11	10.0	0.027	10.0	0.45 ± 0.05
12	10.0	0.043	10.0	0.33 ± 0.03
13	10.0	0.035	1.6	0.39 ± 0.04
14	10.0	0.035	18.4	0.37 ± 0.04
15 (C) **	10.0	0.035	10.0	0.42 ± 0.04
16 (C)	10.0	0.035	10.0	0.41 ± 0.04

* parameter value ± standard deviation, ** (C)—center of plan.

**Table 4:** Analysis of the experiments findings—the initial assessment of the effects by Statistica 13.

Parameter	Evaluation of Effects, Zn, mg/L, R^2^ = 0.9421, R^2^_adj_ = 0.8552, 3 Parameter, 1 Block, 16 Experiments, MS = 0.0336								
	Effect	Standard Error	*p*-Value *	−95% Confidence Interval	+95% Confidence Interval	Factor	Standard Error of Factor	Lower Confidence Interval	Upper Confidence Interval
Constant value	3.396	0.129	0.0222	0.079	0.712	0.396	0.129	0.079	0.712
pH (L) **	−0.800	0.099	0.0002	−1.043	−0.557	−0.400	0.050	−0.521	−0.279
pH (Q) ***	0.549	0.121	0.0039	0.254	0.844	−0.274	0.060	0.127	0.422
V Na_2_CS_3_ (L)	−0.242	0.099	0.0507	−0.485	0.001	−0.121	0.050	−0.242	0.001
V Na_2_CS_3_ (Q)	0.075	0.121	0.5555	−0.220	0.370	0.038	0.060	−0.110	0.185
Time (L)	−0.036	0.099	0.7316	−0.279	0.207	−0.018	0.050	−0.139	0.104
Time (Q)	0.068	0.121	0.5923	−0.227	0.363	0.034	0.060	−0.113	0.182
**** pH (L) relative to V Na_2_CS_3_ (L)	0.208	0.130	0.1608	−0.110	0.525	0.104	0.065	−0.055	0.262
**** pH (L) relative to Time (L)	0.008	0.130	0.9558	−0.310	0.325	0.004	0.065	−0.155	0.162
**** V Na_2_CS_3_ (L) relative to Time (L)	−0.007	0.130	0.9558	−0.325	0.310	−0.004	0.065	−0.162	0.155

* statistically significant if *p* < 0.05, ** L—linear effect, *** Q—quadratic effect, **** (L) relative to (L)—linear combination of the independent parameters.

**Table 5:** Analysis of the experiments’ findings after eliminating statistically insignificant linear interactions ((L) relative to (L)) of the parameters—final assessment of the effects by Statistica 13.

Parameter	Evaluation of Effects, Zn, mg/L, R^2^ = 0.9173, R^2^_adj_ = 0.8622, 3 Parameter, 1 Block, 16 Experiments, MS = 0.0320									
	Effect	Standard Error	*p*-Value *	−95% Confidence Interval	+95% Confidence Interval	Factor	Standard Error of Factor	Lower Confidence Interval	Upper Confidence Interval	
Constant value	0.396	0.126	0.01199	0.110	0.681	0.396	0.126	0.110	0.681	
pH (L) **	−0.800	0.097	0.00002	−1.019	−0.581	−0.400	0.048	−0.510	−0.290	
pH (Q) ***	0.549	0.118	0.00117	0.283	0.815	0.274	0.059	0.141	−0.408	
V Na_2_CS_3_ (L)	−0.242	0.097	0.03400	−0.461	−0.023	−0.121	0.048	−0.231	−0.011	
V Na_2_CS_3_ (Q)	0.075	0.118	0.53829	−0.191	0.341	0.038	0.059	−0.095	0.171	
Time (L)	−0.036	0.097	0.72111	−0.255	0.183	−0.018	0.048	−0.127	0.092	
Time (Q)	0.068	0.118	0.57641	−0.198	0.334	0.034	0.059	−0.099	0.167	

* statistically significant if *p* < 0.05, ** L—linear effect, *** Q—quadratic effect.

**Table 6:** Analysis of the experiments’ findings by Statistica 13—verification of the adequacy of the model by ANOVA.

Parameter	Evaluation of Effects, Zn, mg/L, R^2^ = 0.9173, R^2^_adj_ = 0.8622, 3 Parameter, 1 Block, 16 Experiments, MS = 0.0320			
	SS ***	**** MS	***** F	*p*-Value
pH (L) *	2.185	2.185	68.222	0.000017
pH (Q) **	0.698	0.698	21.794	0.001171
V Na_2_CS_3_ (L)	0.200	0.200	6.238	0.033997
V Na_2_CS_3_ (Q)	0.013	0.013	0.409	0.538292
Time (L)	0.004	0.004	0.136	0.721105
Time (Q)	0.011	0.011	0.336	0.576406
Error	0.288	0.032	–	–

* L—linear effect, ** Q—quadratic effect, *** SS—predicted residual error of sum of squares, **** MS—mean square error, ***** F statistics.

**Table 7:** Calculated linear (L) and quadratic (Q) coefficients of the fitted model by Statistica 13.

Parameter		Regression Coefficients, R^2^ = 0.9173, R^2^_adj_ = 0.8622, 3 Parameter, 1 Block, 16 Experiments, MS = 0.0320				
	Regression Coefficient	*** SE	t-Value **** df = 9	95% Confidence Interval Lower Limit	95% Confidence Interval Upper Limit	***** *p*-Value
Intercept	34.707	7.592	4.572	17.533	51.881	0.001344
pH (L) *	−5.890	1.177	−5.004	−8.552	−3.227	0.000735
pH (Q) **	0.274	0.059	4.668	0.141	0.408	0.001171
V Na_2_CS_3_ (L)	−129.512	164.921	−0.785	−502.589	243.564	0.452454
V Na_2_CS_3_ (Q)	1504.599	2351.944	0.640	−3815.868	6825.066	0.538292
Time (L)	−0.031	0.048	−0.642	−0.139	0.078	0.536893
Time (Q)	0.001	0.002	0.580	−0.004	0.007	0.576406

* L—linear effect, ** Q—quadratic effect, *** SE—standard error, **** df—degree of freedom, ***** statistically significant if *p* < 0.05.

**Table 8:** The concentration of Zn in treated wastewater after RSM application in optimal conditions (pH = 10.75 ± 0.10, V Na_2_CS_3_ dose = 0.043 mL/L and time = 5, 10, 15 and 20 min—experimental model verification).

Parameter	Zn, mg/L after 5 Min	Zn, mg/L after 10 Min	Zn, mg/L after 15 Min	Zn, mg/L after 20 Min
Concentration of Zn, predicted	0.20	0.15	0.17	0.25
Concentration of Zn, experimental	0.15 ± 0.03	0.21 ± 0.03	0.20 ± 0.02	0.20 ± 0.02

**Table 9:** Selected physicochemical parameters of treated galvanic wastewater after RSM application (optimal conditions, pH = 10.75 ± 0.10, V Na_2_CS_3_ dose = 0.043 mL/L and time = 5 min).

Parameter	Unit	Result *	Effect (%) **
pH	-	10.75 ± 0.05	-
Specific electrical conductivity (SEC)	mS/cm	20.61 ± 2.06	↑ 1.48
Salinity	g NaCl/L	12.02 ± 1.20	↑ 18.31
Turbidity	FAU	<10	-
Color	mg Pt/L	<10	-
Total Suspended Solids (TSS)	mg/L	4 ± 1	↓ 68.00
Chemical Oxygen Demand (COD)	mg O_2_/L	2860 ± 143	↓ 1.38
Total Organic Carbon (TOC)	mg/L	930 ± 47	↓ 5.58
Chloride	mg/L	6250 ± 313	↓ 0.79
Sulphate	mg/L	62 ± 3	↓ 4.62
Total phosphorus (Total P)	mg/L	<0.3	< 0.3
Total nitrogen (Total N)	mg/L	32.0 ± 2	↓ 13.51
Complexing compounds (calculated as EDTA)	mg/L	69 ± 4	↓ 1.43
Zinc (Zn)	mg/L	0.15 ± 0.03	↓ 99.99
Copper (Cu)	mg/L	<0.05	-
Nickel (Ni)	mg/L	0.074 ± 0.001	↓ 67.83
Cadmium (Cd)	mg/L	<0.05	-
Lead (Pb)	mg/L	<0.05	-
Iron (Fe)	mg/L	0.115 ± 0.003	↓ 88.61
(undiluted) ASAT *** (dehydrogenase activity)	%		−70 (±10)
(1:10) ASAT *** (dehydrogenase activity)	%		−15 (±10)

* parameter value ± the measurement uncertainty for an extension factor k = 2, if applicable ** Effect = $\frac{\left(C1-C2\right)x100\%}{C1}$ and Effect = $\frac{\left(C2-C1\right)x100\%}{C1}\;$ (for parameters whose values have increased), where c_1_—concentration in raw galvanic wastewater, c_2_—concentration in treated galvanic wastewater, ↑—increase in the parameter value, ↓—decrease in the parameter value, *** Activated Sludge Activity Test; negative results compared to reference value indicate a significant inhibition of the dehydrogenase activity by the sample.

**Table 10:** Results of one-way ANOVA test for phytotoxicity tests.

Tasted Plant	Average Length	SS	df	MS	F	*p*
*Sinapis alba*	Root	3.35	27	0.12	55.41	<0.001
	Shoot	9.04	27	0.08	112.90	<0.001
*Lepidium sativum*	Root	1.39	27	0.05	328.54	<0.001
	Shoot	1.40	27	0.05	261.37	<0.001

## Data Availability

Not applicable.
